# Telling friend from foe in emergency vertigo and dizziness: does season and daytime of presentation help in the differential diagnosis?

**DOI:** 10.1007/s00415-020-10019-x

**Published:** 2020-07-11

**Authors:** Klaus Jahn, Antoanela Kreuzpointner, Thomas Pfefferkorn, Andreas Zwergal, Thomas Brandt, Andreas Margraf

**Affiliations:** 1 German Center for Vertigo and Balance Disorders (DSGZ), Ludwig-Maximilians University of Munich, Klinikum Grosshadern, Marchioninistrasse 15, 81377 Munich, Germany; 2 Department of Neurology, Ludwig-Maximilians University of Munich, Munich, Germany; 3 Clinical Neurosciences, Ludwig-Maximilians University of Munich, Munich, Germany; 4 Institute for Medical Education (DAM), Ludwig-Maximilians University of Munich, Munich, Germany; 5 Department of Anesthesiology, Intensive Care Medicine and Pain Therapy, University Hospital Muenster, Muenster, Germany; 6 Department of Neurology, Klinikum Ingolstadt, Ingolstadt, Germany; 7 Department of Neurology, Schoen Clinic Bad Aibling, Bad Aibling, Germany

**Keywords:** Vertigo, Dizziness, Emergency room, Benign paroxysmal positional vertigo, Stroke

## Abstract

Distinguishing between serious (e.g., stroke) and benign (e.g., benign paroxysmal positional vertigo, BPPV) disorders remains challenging in emergency consultations for vertigo and dizziness (VD). A number of clues from patient history and clinical examination, including several diagnostic index tests have been reported recently. The objective of the present study was to analyze frequency and distribution patterns of specific vestibular and non-vestibular diagnoses in an interdisciplinary university emergency room (ER), including data on daytime and season of presentation. A retrospective chart analysis of all patients seen in a one-year period was performed. In the ER 4.23% of all patients presented with VD (818 out of 19,345). The most frequent-specific diagnoses were BPPV (19.9%), stroke/transient ischemic attack (12.5%), acute unilateral vestibulopathy/vestibular neuritis (UVH; 8.3%), and functional VD (8.3%). Irrespective of the diagnosis, the majority of patients presented to the ER between 8 a.m. and 4 p.m. There are, however, seasonal differences. BPPV was most prevalent in December/January and rare in September. UVH was most often seen in October/November; absolute and relative numbers were lowest in August. Finally, functional/psychogenic VD was common in summer and autumn with highest numbers in September/October and lowest numbers in March. In summary, daytime of presentation did not distinguish between diagnoses as most patients presented during normal working hours. Seasonal presentation revealed interesting fluctuations. The UVH peak in autumn supports the viral origin of the condition (vestibular neuritis). The BPPV peak in winter might be related to reduced physical activity and low vitamin D. However, it is likely that multiple factors contribute to the fluctuations that have to be disentangled in further studies.

## Introduction

Patients presenting to the emergency room (ER) must be diagnosed quickly to identify those needing urgent treatment [35]. The diagnostic algorithm varies according to the leading symptoms upon presentation and possible differential diagnoses. Vertigo and dizziness (VD) are among the most frequent symptoms reported by patients at ER consultations. In neurological ER services, VD accounts for > 10% of patients and is the third most common leading symptom after headache (20%) and motor deficit (13%) [27], respectively, after cerebrovascular disorders (28%) and headache (22%) [5]. VD may present as acute vestibular syndrome (AVS) in unilateral vestibular dysfunction (peripheral or central), but other diagnoses have to be kept in mind from benign paroxysmal positional vertigo (BPPV) to anxiety related dizziness [29]. Patients presenting to the ER are diverse with regard to presenting symptoms, underlying pathologies, pre-existing conditions, age and socioeconomic background [10, 30, 32, 34]. The major concern for ER physicians is to differentiate between potential life-threatening central symptoms and disorders that are more benign. Over the last years, algorithms have been suggested to distinguish between posterior circulation stroke and vestibular neuritis in AVS, i.e., in patients presenting with rotational vertigo, horizontal-rotatory spontaneous nystagmus, postural imbalance with directional falls, nausea, and vomiting. For clinical evaluation, the HINTS procedure (head impulse test, gaze evoked nystagmus, skew deviation) is now widely applied [11] but its usability is still under discussion [15, 26]. In addition, there is growing evidence for predictive factors in non-AVS VD [35]. For the current study we asked the following questions: (1) which patient groups present to the ER and how does the spectrum of disorders differ between ER and a tertiary care outpatient dizziness clinic?; (2) how is the usage of diagnostic/imaging procedures to differentiate between benign and serious causes of VD?; and (3) does the seasonal and circadian distribution of presentation differ between diagnoses? To address these questions, we performed a retrospective chart analysis of all non-surgical patients presenting in a 1-year period to the ER of a tertiary care university hospital in Germany. We hypothesized to see (1) more AVS patients compared to a tertiary care dizziness clinic in the ER; (2) a high number of diagnostic imaging without any pathologic correlate; and (3) a peak of benign paroxysmal positional vertigo (BPPV) in morning hours, presentation of stroke/transitoric ischemic attack (TIA) without specific circadian peaks, and a seasonal preponderance of vestibular neuritis (UVH) in spring and autumn because of the suspected viral etiology.

## Methods

### Patient inclusion, screening and data acquisition

A retrospective chart analysis was performed at the interdisciplinary emergency department of the Ludwig-Maximilians-University of Munich (LMU, Klinikum Grosshadern). Non-surgical admissions between December 31, 2009 and January 01, 2011 were screened (total patient count: 19,345) and all patients that received the label “VD” (German: “Schwindel”) at the ER counter were included in the study (*n* = 938). Data (gender, age, day and time of admission, medical disciplines involved, tests performed, final diagnosis, inpatient/outpatient status) were entered into an Excel sheet (Microsoft). Further data (clinical findings, test results, and final diagnosis after inpatient workup) were acquired through patient’s charts and dismissal letters. Data were numerically coded for subsequent calculations. The results were matched with data of the tertiary care dizziness clinic at the “German Center for Vertigo and Balance Disorders” in Munich, Germany (same time period, *n* = 1667). Table 1 shows the definition of diagnostic groups. As diagnoses were made in a clinical environment, they are not based on formal diagnostic criteria. The study has been performed in accordance with the ethical standards laid down in the 1964 Declaration of Helsinki and its later amendments.

**Table 1:** Clinical diagnostic criteria in the emergency room

Diagnosis	Definition
Benign paroxysmal positional vertigo (BPPV)	Transient vertigo triggered by change of head position in relation to gravity
Bilateral vestibular hypofunction (BVH)	Dizziness and imbalance during walking with worsening in darkness/with eyes closed; bilateral pathological head impulse test
Menière’s disease (MD)	Transient VD with hearing loss or other ear-related symptoms and a history of at least one other similar attack
Orthostatic VD	Dizziness related to orthostatic challenges (getting up from a lying or sitting position) with pathological orthostasis test (drop of systolic blood pressure > 20 mmHg within 3 min after changing into an upright position)
Perilymph fistula (PF)	VD triggered by coughing, sneezing; and pathological fistula signs (nystagmus with Valsalva maneuver or tragus pressure)
Persistent postural-perceptual dizziness; Functional/psychogenic/phobic VD (PPPD):	Transient or persistent dizziness or imbalance without abnormal findings on clinical examination; dissociation between subjective VD and objective findings
Stroke/TIA:	Acute vestibular syndrome (spontaneous nystagmus, nausea and/or vomiting, and postural imbalance) or other VD presentation caused by a stroke or a transient ischemic attack (symptoms < 24hours), with or without additional neurological signs (e.g., double vision, dysarthrophonia, hemiparesis). Diagnosis was confirmed either by MRI and/or by the typical clinical presentation (positive HINTS test, clinical findings)
Vestibular migraine (VM)	VD with migraine headache, sensitivity to light/noise and with a history of at least one similar attack. For attacks without headache: history of typical migraine and at least two similar attacks
Acute unilateral vestibular hypofunction/vestibular neuritis (UVH)	Acute vestibular syndrome (spontaneous nystagmus, nausea and/or vomiting, and postural imbalance) compatible with peripheral loss of function (HINTS with pathological head impulse, no skew, no direction changing nystagmus). No additional focal neurological signs
Vestibular paroxysmia (VP)	At least 5 short attacks of VD (seconds to minutes) not exclusively related to head position changes in relation to gravity
VD of other origin (others)	Any VD secondary to a variety of disorders not related to balance control (e.g., arterial hypertension, viral pneumonia, metabolic changes)
VD of unknown origin/unspecified (unclear):	Any VD not fulfilling criteria of the other groups and/or classified as “VD of unknown origin” at ER dismissal. This also includes posttraumatic VD without peripheral or central vestibular signs (normal head impulse and no ocular motor signs)

Routine diagnostic workup included: vital signs, blood pressure, pulse, temperature, blood oxygen saturation, laboratory workup, neurologic/physical examination, electrocardiogram. Dependent on clinical presentation and differential diagnosis a proportion of patients received the following tests: computed tomography (CT), magnetic resonance imaging (MRI), vestibular testing (calorics, head impulse tests, evoked potentials, subjective visual vertical, orthoptic evaluation), stabilometry, ultrasound of brain vessels, electroencephalography, lumbar puncture, nerve conduction studies, orthostasis tests (Schellong test). For grouped assessment of daytime presentation peaks, the 24 h of the day were split up into 4-h periods: 0:01–4 a.m., 4:01–8 a.m., 8:01-noon, 12:01–4 p.m., 4:01–8 p.m., 8:01-midnight.

### Statistical analysis

Statistical calculations were performed utilizing SPSS V16.0 (SPSS Inc, Chicago, USA), GraphPad Prism version 6.01 (GraphPad Software Inc, San Diego, USA), and SigmaPlot version 12.0 (Systat, Erkrath, Germany). Correlation analysis was performed using the Pearson’s R coefficient. Statistical comparison of two groups was performed using *t* test, multi-group comparisons were performed using a one-way ANOVA or ANOVA on ranks, respectively (Bonferroni correction for multiple testing). Calculation of diagnostic effectiveness/accuracy was performed by creating the sum of all precise diagnostic groups divided by the total amount of diagnosed dizziness cases (including imprecise diagnoses or vertigo of unknown origin and discharge without documented diagnosis). *P* < 0.05 was considered statistically significant. Data are presented as mean ± standard deviation (SD) or ± standard error of the mean (SEM) as stated.

## Results

19,345 patients were assessed for occurrence of VD symptoms. Out of these, 938 patients were included in the primary analysis. 120 patients had to be removed from further analysis due to lack of discharge letters or due to lack of VD symptoms on second look (e.g., nausea instead of VD). 818 patients (4.23% of 19,345) presented with VD (60.6% female, age 51.9 ± 19.3 years, range 3–103 years). Patients had been primarily assigned to the neurologist (88.2%; 18.5% of all neurology ER patients), the otolaryngologist (7.2%), or other disciplines (4.6%). 658 patients presented with primary VD, 280 with VD secondary to other conditions. From the primary vertigo population, 205 patients (31.2%) were admitted to the hospital after ER evaluation. 81.7% of patients with stroke/TIA or UVH were admitted to inpatient care, whereas PPPD and BPPV remained primarily outpatient (10.6% inpatients; Table 2).

**Table 2:** Relative frequencies of diagnoses of patients presenting with VD

Diagnosis	Outpatient Clinic (%)	Emergency Room (%)	Female (%)	mean age (years)	admitted to ward (%)
BPPV	18.5	19.9	60.7	57.1 ± 17.5	16.8
CV	9.2	12.5	52.8	64.7 ± 14.9	81.7
PPPD	17.9	8.3	56.5	40.7 ± 16.7	10.6
UVH	9.5	8.3	59.2	53.3 ± 15.4	70.2
VM	13.0	7.0	74.4	42.2 ± 18.8	37.5
MD	9.8	6.0	54.8	55.6 ± 17.4	58.8
BVH	9.8	0.5	66.6	51.4 ± 19.3	33.3
VP	2.9	0.4	50.0	49.8 ± 19.1	50.0
PF	0.7	0.2	0.0	54	100.0
others	5.2	14.6	68.2	51.9 ± 19.4	42.2
unknown	3.5	22.5	63.2	51.8 ± 19.3	13.3

The second column shows relative frequencies for the tertiary care outpatient clinic (dizziness clinic at the University of Munich) in the same period for comparison. All other columns refer to the data from patients presenting to the emergency room (ER). Diagnoses are sorted by the relative frequencies in the ER. Note that absolute numbers for bilateral vestibular hypofunction (BVH = 3), vestibular paroxysmia (VP = 2), and perilymph fistula (PF = 1) are low

*BVH* bilateral vestibular hypofunction, *BPPV* benign paroxysmal positional vertigo, *CV* central vertigo including stroke/TIA, *MD* Menière’s disease, *PF* perilymph fistula, *PPPD* persistent postural-perceptual dizziness including functional/psychogenic/phobic patients, *UVH* unilateral vestibular failure including vestibular neuritis, *VM* vestibular migraine, *VP* vestibular paroxysmia

The most frequent-specific diagnoses were BPPV (19.86%), central VD including stroke/TIA (12.48%), acute unilateral vestibulopathy/vestibular neuritis (UVH, 8.26%), functional/psychogenic/phobic vertigo (PPPD 8.26%), vestibular migraine (VM, 7.03%), and Menière’s disease (MD, 5.98%). Non-vestibular diagnoses, including traumatic, infectious, toxic causes, electrolyte imbalance, tumors, orthostatic reactions, hypertension, cardiogenic causes, accounted for 14.59% of patients. Compared to the outpatient dizziness clinic, VD of unknown origin was more common in ER (22.5% vs. 3.48%; Table 2).

### Circadian, seasonal, sex and age variations in VD diagnoses

We further analyzed age dependency and seasonal variations for common diagnoses. The oldest diagnostic group was the stroke/TIA group (mean age 64.7 ± 14.9 years), the youngest one was the PPPD group (mean age 40.7 ± 16.7 years). When comparing age dependency of time until discharge/ward-admittance in the ER a one-way ANOVA showed a significant difference but post hoc Bonferroni multicomparisons procedure revealed no significant difference between groups. Utilizing the Pearson’s R coefficient, there was only a weak (*r* = 0.1191; *p* = 0.0023) direct correlation between age and time of treatment within the ER. The lowest time until discharge was present in the group of 21–40, the highest in the age group > 80. There was no significant difference in time until discharge or admittance between sexes. When comparing the major benign vs. life-threatening diagnostic group in this study (BPPV vs. stroke/TIA), the time until discharge or admittance was shorter in the BPPV group compared to the stroke/TIA group (*p ≤ 0.05).

**Fig. 1:**
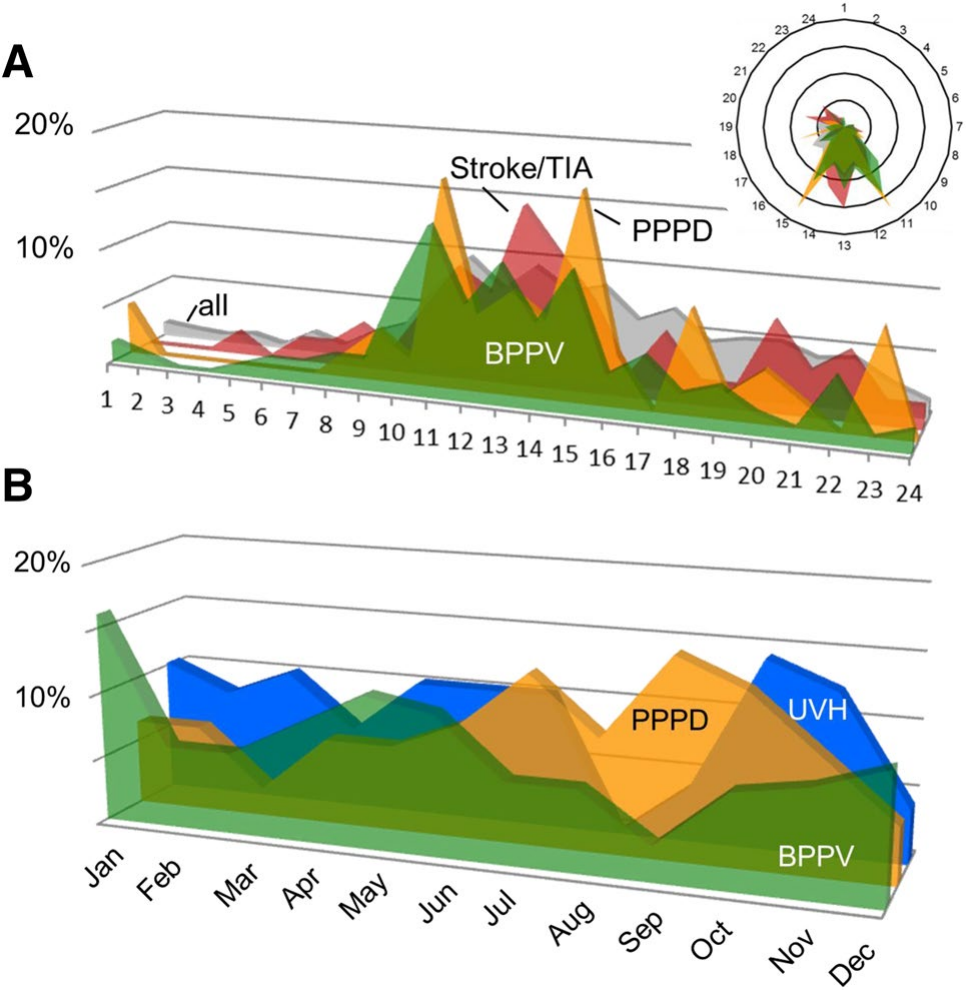
Diagnoses along the day and during the year. **a** Distribution of patients along the 24 h of the day (the same shown in the circular inset). Diagnostic groups as labeled. Note that most patients present during regular working hours. **b** Distribution of patients along the 12 month of the year. Diagnostic groups as labeled. Note the high rate of UVH in autumn and the high rate of BPPV in winter. *BPPV* benign paroxysmal positional vertigo, *PPPD* persistent postural-perceptual dizziness including functional/psychogenic/phobic patients, *UVH* unilateral vestibular hypofunction including vestibular neuritis

Concerning daytime of presentation, there was no difference between diagnostic groups. The majority of patients presented during regular working hours (Fig. 1a). In contrast, there were seasonal differences in presentation for some diagnoses (Fig. 1b). BPPV peaked in December/January, whereas MD (July), VM, PPPD (September) and UVH (October/November) peaked later throughout the year. Whereas PPPD was common in late summer, BPPV and UVH presentations were low in September.

### Number of diagnostic steps depends on causes of VD

**Fig. 2:**
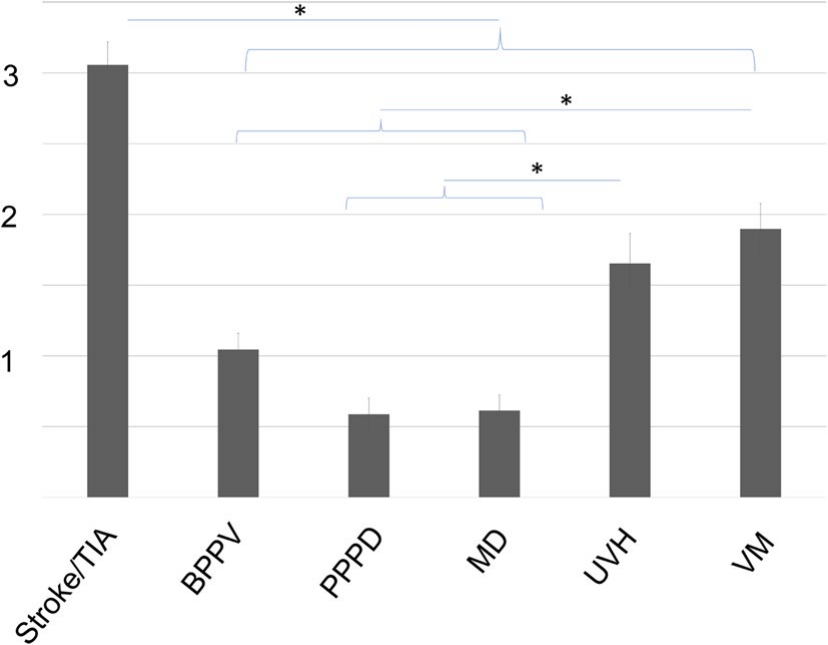
Number of imaging procedures (CT/MRI) per patient in different diagnostic groups. The amount of imaging procedures was highest in the stroke/TIA group, lowest in the PPPD group. (mean ± SEM; *p < 0.05). *BPPV* benign paroxysmal positional vertigo, *MD* Menière's disease, *PPPD* persistent perceptual-postural dizziness, *UVH* unilateral vestibular hypofunction, *VM* - vestibular migraine

The highest number of diagnostic steps (including physical/neurologic examination, laboratory workup or CT imaging) was performed in the stroke/TIA group. The least number of diagnostic measures was taken in the MD group. The number of diagnostic steps was not different between female and male patients (4.1 ± 3.0 vs. 4.1 ± 2.9). The highest number of diagnostic steps per age group was used in the > 80 group (4.7 ± 2.9), the lowest in the group of 21–40 years (3.5 ± 2.6, mean ± SD). For diagnostic workup, the highest number of imaging procedures were performed in the stroke/TIA group (3.1 ± 1.2), whereas a PPPD patient only received 0.6 ± 0.8 imaging procedures (Fig. 2).

Calculation of diagnostic effectiveness/accuracy was performed by dividing number of cases with specific diagnosis by all cases. The diagnostic effectiveness, thus the diagnostic accuracy was 64.1% in the ER, compared to 96.5% in a specialized outpatient clinic. Taking into consideration unspecific diagnoses, the diagnostic effectiveness is even lower with 54.7% in the ED vs. 91.4% in the outpatient clinic.

## Discussion

Over the last years, emergency vertigo and dizziness became a research focus and a number of scores and algorithms have been described to overcome the challenges of making a fast and correct diagnosis [3, 8, 35]. This is crucial, as a substantial proportion of patients will suffer from serious causes of VD, in particular posterior circulation stroke. The retrospective analysis confirms a number of findings from other studies. In addition to that, we asked the specific question if the daytime and season of presentation to the ER contains information on the likelihood of a certain diagnosis. The main finding is that patients with acute VD show up in the ER during normal working hours irrespective of the diagnosis made later on. This most likely reflects the sensible decision of the affected people to wait a few hours if symptoms resolve spontaneously in case they start during the night. We do not have any systematic information on how long symptoms already lasted when patients are admitted to the ER. Some seasonal fluctuations are worth being discussed. Beside this, the data show that in contrast to specialized outpatient centers a large proportion of VD patients get unspecific or descriptive diagnoses. A majority receives brain imaging that is not appropriate.

Our retrospective study confirms the high prevalence of VD in the population and in the ER [22]. We found that almost 5% of all patients presenting to the ER of a large tertiary care university hospital have dizziness symptoms (vertigo/dizziness/lightheadedness) which underlines the importance of appropriate training on the topic for emergency physicians. At the University of Munich about 20% of all ER patients seen by the neurologist suffer from VD. These results are congruent with other reports of about 3.5% VD patients among ER patients [2, 4], respectively, 12–13% of neurology ER patients [5, 27], making it the third most frequent leading symptom in the neurologic ER after headache (20%) and motor deficit (13%). Mean age at presentation in our cohort was 51.7 years. A comparison between VD patients and non-VD patients in the ER showed that VD patients are older (51 vs. 43.7 years) [28]. Sex distribution of VD in our cohort was comparable to published data from the general population [22].

The most frequent diagnoses in our cohort were BPPV, followed by stroke/TIA, PPPD, UVH, VM, and MD. A large number of patients did not receive a specific diagnosis, but a mere descriptive one. This is in accordance with a large-scale study from emergency departments in the US, with symptom diagnoses (22%) being more common among dizziness patients than among other symptom complexes [23]. Others found an even higher incidence of mere descriptive diagnoses (40%) [2]. This stresses the difficulty in making the correct diagnosis and reflects the still prevailing lack of knowledge in non-specialized physicians. In the outpatient vertigo clinic of a tertiary care university hospital (German Center for Vertigo and Balance Disorders), the “unclear” cases were minimal with a rate of 3.5%. BPPV, PPPD, and VM are the most frequent diagnoses in the center. In addition, the diagnostic effectiveness was much higher compared to the ER. This is most likely due to the training of staff and to the high number of dizzy patients treated in the center. The availability and use of basic diagnostic tests was similar in both environments (history taking, physical examination including positioning maneuvers, caloric testing, video-head impulse test, subjective visual vertical).

The majority of patients presented during the day between 8 a.m. and 4 p.m. This shows that patients do not expect stroke and cannot distinguish between serious and benign causes of VD. In particular, they do not interpret the VD symptoms as a possible stroke sign, as this should lead to immediate presentation to the ER. Obviously, patients in some cases waited for normal day time before they decided to visit the ER. It is necessary to increase the alertness of the general population that VD can be symptoms of acute stroke.

BPPV patients in our study presented more often during December and January compared to summer months. This could be due to vitamin D deficiency in winter. A similar seasonal preponderance has been reported before and related to vitamin D, although the month with highest prevalence of BPPV were March and April in this study [20]. However, the relationship between BPPV and low vitamin D could not be confirmed in meta-analyses [1]. An alternative explanation could be the lack of physical activity in winter as BPPV occurs most often after prolonged rest (e.g., in the morning). Furthermore, BPPV incidence is increased after inflammatory ear disorders such as vestibular neuritis which is common in autumn as our study confirms. It is difficult to tell why functional VD (PPPD) seems more common in late summer (September). The seasonal fluctuation might in part just reflect usage of the health care system after summer holydays or similar factors not related to the disorder itself. However, it is well known that mental and behavioral disorders show seasonal fluctuation. Affective disorders are more common in winter and it has been shown that somatic symptoms can precede the affective nadir in autumn [14, 25]. It is another interesting finding that emergency room admissions related to mental and behavior disorders increase after very high temperatures in summer (peak 7 days after T > 28 °C; [33]). However, the summer 2010 was not unusual hot in Germany.

In contrast to the outpatient setting in a tertiary care unit, the causes in ER are more likely to be life-threatening (acute stroke/TIA: > 10% vs. 0% for ER vs. specialized outpatient clinic). Thus, it is of utmost importance to adequately discern benign from life-threatening conditions within a short period of time (“time is brain”). As often stated, the differentiation between vestibular neuritis (UVH) and posterior circulation stroke can be difficult, especially when a stroke presents with isolated dizziness/vertigo, without other focal neurological symptoms [6, 7, 9, 12, 13]. For the acute vestibular syndrome (AVS), the rate of peripheral (UVH) and central causes (stroke/TIA) in our study was similar (12.48% resp. 8.26%). Stroke/TIA-rates among ER patients reported in the literature range between 3.2% up to 18% [2, 12, 24]. Cerebellar infarctions can present as AVS (“pseudoneuritis”). The same is true for some cases of isolated nodulus infarction and isolated vestibular nucleus infarction [13, 17, 18]. The HINTS test helped a lot to reduce the rate of misdiagnosis in the AVS population with 100% sensitivity and 96% specificity for stroke identification [11, 26]. Several factors favor a central/stroke diagnosis. In a prospective observational study with 413 ER patients with VD symptoms, the following predictive factors for stroke have been determined: 65 years of age, ataxia symptoms, presence of focal neurological symptoms, history of previous stroke and diabetes mellitus [2]. Kerber et al. found stroke patients to be significantly older and predominantly male compared to non-stroke dizziness patients (stroke vs. non-stroke: mean 69 vs. 65 years and 55% vs. 36% males), having other neurologic symptoms and at least two of the risk factors: hypertension, history of coronary artery disease, diabetes, previous TIA/stroke, current smoker, hypercholesterolemia, and atrial fibrillation [12]. In agreement in our study, the stroke/TIA group contained the highest number of male patients and the highest mean age (64.7 years). As a bedside tool to identify cerebrovascular causes of dizziness, a score regularly applied for prediction of risk of stroke after TIA can be utilized, namely the ABCD2 score (age ≥ 60 years; blood pressure ≥ 140/90; clinical features; duration of symptoms and diabetes) [21]. Having these risk factors in mind can facilitate identifying serious central causes of vertigo. Nonetheless, it is important not to diminish one’s clinical alertness for unusual cases with a low ABCD2 score, as for example, our stroke/TIA group also contained a 26-year-old patient.

Regarding the functional/psychogenic/phobic dizziness (PPPV), we found a lower prevalence in the ER (about 8%) compared to the specialized outpatient clinic (18%). It is remarkable that still a large number of these patients present to the ER. Overall, our study exemplifies that profound knowledge of six principal VD-related diagnoses (BPPV, PPPD, VM, MD, stroke/TIA, UVH) can diagnose more than 50% of all patients presenting with VD symptoms in the ER. The high number of “unclear” diagnoses shows the need for further educational endeavors and optimized diagnostic algorithms to improve the diagnostic effectiveness within the ER. Therefore, thorough training of ED physicians in the field of dizziness symptoms and its examination techniques (e.g., HINTS) is indispensable to better diagnose underlying causes [6]. Importantly, the frequency of VD in the ER and the large proportion of serious causes (stroke/TIA) stress the importance of thorough training routines for neurologists and otolaryngologists working in the ER. Recent work in a Canadian cohort showed that there are major training deficits in final year otolaryngologists with regard to vertigo diagnostic testing, more specifically the bedside head impulse test [16, 19]. A survey-based study emphasized interdisciplinary differences between otolaryngologists and non-otolaryngologists with regard to vertigo diagnosis and treatment practices [24]. Interdisciplinary thinking between neurology, ENT, and internal medicine, is much needed not only to appropriately detect, diagnose and treat life-threatening causes of VD, but also with regard to the high number of unspecified diagnoses termed, demanding an interdisciplinary, holistic view of dizzy patients.

VD is responsible for major costs within both the emergency department and the inpatient setting [16]. Resource use has been found to be disproportionally high for ER patients with VD symptoms, especially with regard to diagnostic imaging [24]. As could be seen from the HINTS-studies, clinical testing shows a better detection rate of strokes in acute AVS than cranial imaging (MRI or CT) [11]. Of interest, another four-element algorithm termed “STANDING” (spontaneous and positional nystagmus, evaluation of nystagmus direction, head impulse test, evaluation of equilibrium) was shown to offer good reliability and high accuracy in the exclusion of stroke and other life-threatening causes of VD even for non-sub-specialists in the ER [31]. In the specialized outpatient clinic of the German Center for Vertigo and Balance Disorders, only a limited number of patients (about 4%) receive CT or MRI scans (e.g., in perilymph fistula and vestibular paroxysmia). This is in contrast to the high number of cranial imaging of ER patients (approx. 70% in our study), still leaving 22% of patients without a precise diagnosis, in contrast to only 3% of unclear cases in the outpatient clinic. CT-scans generally have a low diagnostic value for acute ischemic infarctions in the posterior cranial fossa [28]. We, therefore, plead for a specific, reasonable appliance, adapted to the individual diagnoses and only following adequate clinical testing. Interestingly, our study exemplifies that reaching the diagnosis for a life-threatening condition (stroke/TIA) requires most time. Reasons for this could be the diagnostic burden involved (required cranial imaging) or diagnostic uncertainty, which by usage of adequate training of the aforementioned algorithms might be reduced.

Our study has some limitations. The retrospective character limits the accuracy of the data available. For a number of patients (excluded from analysis), a discharge letter was not available. Diagnoses depend on the experience of the experts and ER physicians involved. Documentation was not standardized, and diagnostic criteria could not be applied. Additionally, this study did not include multicenter data but is based on data from a single center (the ER and a specialized outpatient clinic of a single tertiary care university hospital).

## Conclusions

In our cohort, the probability of stroke and serious causes of VD was high. For a subgroup of patients, the test protocols and scoring systems (HINTS, ABCD2 score) are crucial for discernment between stroke and more benign disorders. Stroke patients with VD as the chief complaint present to the ER during regular working hours which points to a lack of alertness for VD being a red flag symptom. The large number of patients that received a purely descriptive diagnosis implies a lack of knowledge and training in the field. A Knowing-Training-Transferring (KTT) approach for ED physicians which implies the knowledge of most common VD diagnoses as presented in this study would be needed to ensure adequate training of algorithms and clinical testing and better interdisciplinary networking for transferal of unclear cases to appropriate dizziness centers to avoid unnecessary hospitalizations and misdiagnoses. Future endeavors are aimed at establishing machine learning tools as supportive elements for VD-related diagnostics, whereas implementation of such assets must be evaluated in detail and with caution.
